# Enhanced Electron–Phonon Coupling of Superconductivity in Indium-Doped Topological Crystalline Insulator SnTe

**DOI:** 10.3390/ma19010073

**Published:** 2025-12-24

**Authors:** Kwan-Young Lee, Gareoung Kim, Jae Hyun Yun, Jin Hee Kim, Jong-Soo Rhyee

**Affiliations:** Department of Applied Physics, G-LAMP NEXUS Institute, Kyung Hee University, Yongin 17104, Republic of Korea; andyky@khu.ac.kr (K.-Y.L.); noah04@naver.com (G.K.); ataxtr@khu.ac.kr (J.H.Y.); jinheekim@khu.ac.kr (J.H.K.)

**Keywords:** topological crystalline insulator, s-wave superconductor, topological superconductor, weak-to-strong coupling transition, SnTe

## Abstract

Indium-doped SnTe (Sn_1−x_In_x_Te) provides a model platform for exploring the emergence of superconductivity within a topological crystalline insulator. Here, we present a systematic investigation of the structural, transport, and thermodynamic properties of high-quality single crystals with 0.0 ≤ x ≤ 0.5. All compositions up to x = 0.4 form a single-phase cubic structure, enabling a controlled study of the superconducting state. Electrical resistivity and specific heat measurements reveal a bulk, fully gapped s-wave superconducting phase whose transition temperature increases monotonically with In concentration, reaching *Tc* ≈ 4.7 K at x = 0.5. Analysis of the electronic specific heat and McMillan formalism shows that the electron–phonon coupling constant *λ_el__-__ph_* systematically increases with doping, while the Debye temperature systematically decreases, resulting in the lattice softening. This behavior, together with the observed evolution of the normal-state resistivity exponent from Fermi-liquid (*n* ≈ 2.04) toward non-Fermi-liquid values (*n* ≈ 1.72), demonstrates a clear crossover from weak to strong interaction with increasing In content. These results establish Sn_1−x_In_x_Te as a tunable superconducting system in which coupling strength can be continuously controlled, offering a promising platform for future studies on the interplay between phonon-mediated superconductivity and crystalline topological band structure.

## 1. Introduction

Topological materials have become a central focus of contemporary condensed matter research due to their symmetry-protected electronic states and unconventional quantum phenomena. Among these, topological insulators (TIs) and, more recently, topological crystalline insulators (TCIs) represent classes of materials in which nontrivial boundary states emerge as a consequence of either inversion- or specific crystal symmetries [1,2,3]. The possibility of combining these topological states with superconductivity has stimulated intense interest because such systems may host exotic quasiparticles, including Majorana fermions, that are highly relevant for fault-tolerant quantum computation [4,5,6].

Early experimental progress in this direction was demonstrated in the copper-intercalated topological insulator Cu_x_Bi_2_Se_3_, which established a pathway to induce superconductivity in materials already possessing topological surface states [7,8,9]. A related and particularly promising platform is SnTe, a prototypical TCI whose topological nature originates from mirror-symmetry-protected Dirac surface states on high-symmetry planes of the rock-salt lattice [10,11,12,13]. Also, SnTe is known as a promising thermoelectric material. The band engineering can maximize the power factor and thermoelectric figure-of-merit zT [14,15,16,17]. Band engineering of SnTe is also important for the topological properties. Band inversion near the L points of the Brillouin zone underpins its nontrivial topology, a mechanism also shared by the Pb_1−x_Sn_x_Te alloy series [13,18]. Given its simple crystal structure and well-understood topological features, SnTe provides a clean starting point for investigating the interplay between topology and emergent superconductivity.

Chemical doping of SnTe with indium has been shown to induce bulk superconductivity, and the Sn_1−x_In_x_Te system has therefore emerged as a candidate material platform for realizing topological superconductivity [19,20,21,22,23,24]. However, despite extensive experimental efforts, the microscopic nature of the superconducting state remains debated. Early studies suggested that superconductivity in In-doped SnTe arises from a conventional weak-coupling electron–phonon mechanism, whereas subsequent reports have hinted at an evolution toward stronger coupling as the indium concentration increases [21,25]. Establishing whether a systematic progression from weak to strong coupling occurs is thus essential for understanding both the superconducting pairing mechanism and the possible emergence of unconventional or topological superconducting states.

In this work, we address this open question through a comprehensive investigation of high-quality Sn_1−x_In_x_Te single crystals (0.0 ≤ x ≤ 0.5). By combining temperature-dependent resistivity, magnetization, and specific heat measurements, we uncover clear and consistent evidence for a doping-driven evolution of electron–phonon coupling. Detailed analysis of the thermodynamic parameters using the McMillan formalism reveals an increasing electron–phonon coupling constant *λ_el__-__ph_* with doping, while the Debye temperature systematically decreased, indicating a phonon softening. Concurrently, the normal-state resistivity exponent deviates systematically from the Fermi-liquid value of *n* = 2, decreasing to *n* ≈ 1.72 at higher doping levels. Together, these results demonstrate a continuous crossover from weak-coupling to strong-coupling superconductivity as indium concentration increases.

By establishing this coupling-strength evolution in a topological crystalline insulator, our study provides an essential foundation for future investigations into whether strong electron–phonon interactions or enhanced density-of-states effects may promote nontrivial superconducting states in Sn_1−x_In_x_Te. More broadly, our results position this material system as a tunable platform for exploring the interplay between topology, electron–phonon interactions, and superconductivity.

## 2. Materials and Methods

Bulk single crystals of Sn_1−x_In_x_Te with nominal indium concentrations from x = 0.0 to 0.5 were synthesized using a Sn-flux method using excessive Sn as a flux. Polycrystal samples were synthesized as precursors using high-purity elements of Sn (99.999%, RND Korea, Gwangmyeong-si, Republic of Korea), Te (99.999%, RND Korea, Gwangmyeong-si, Republic of Korea), and In (99.99%, RND Korea, Gwangmyeong-si, Republic of Korea) with stoichiometric molar ratios of Sn_1−x_In_x_Te (x = 0.0, 0.1, 0.2, 0.3, 0.4, and 0.5). The elements were loaded and sealed in quartz ampoules under vacuum into a quartz ampoule (~10^−4^ Torr). The ampoules were heat treated at 950 °C for 12 h. The polycrystals and Sn flux were loaded in alumina crucible with a volume ratio of Sn–polycrystals = 9:1 and sealed in evacuated quartz ampoules. The high Sn content ensures the mixture is fully liquid at manageable temperatures and can provide a large volume of flux for crystals to grow congruently. The ampoule was heated to 950 °C for 12 h to ensure a homogeneous melt, then slow-cooled to 550 °C over 40 h to promote crystal growth, followed by centrifugation to separate Sn flux. For post-characterization, dilute hydrochloric acid (HCl) was used, as Sn dissolves easily in HCl, while SnTe is relatively stable for short-term exposure. After that the crystals were rinsed with ethanol right after short exposure to acid so as not to damage the surface of crystals.

Structural analysis was performed on powdered samples using powder X-ray diffraction (XRD) with Cu Kα radiation (D8 Advance, Bruker, Berlin, Germany). The diffraction patterns were collected over appropriate angular ranges to identify phase purity and determine structural parameters. For physical property measurements, the crystals were prepared into specific geometries: rectangular specimens for resistivity and samples with a flat bottom for specific heat measurements. All transport and thermodynamic measurements, including temperature-dependent resistivity (2–300 K) using the four-probe method, magnetization, and specific heat, were carried out using a Dynacool Physical Properties Measurement System (PPMS, Quantum Design, San Diego, CA, USA).

## 3. Results and Discussion

### 3.1. Crystal Structure Analysis

**Figure 1:**
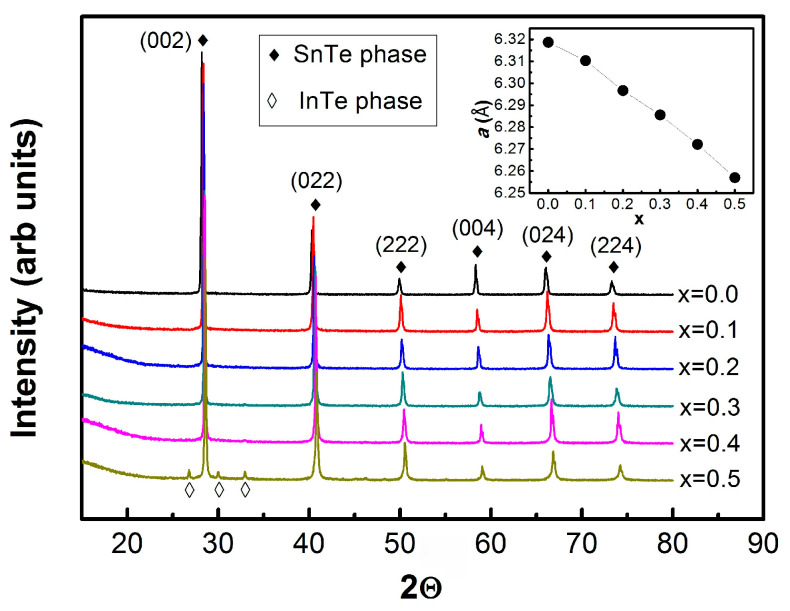
Room-temperature powder X-ray diffraction patterns for the Sn_1−x_In_x_Te sample in the range of x = 0.0–0.5. From x = 0.1 to x = 0.4, the X-ray diffraction pattern shows single phase and over x = 0.5 starts to show secondary phase (tetragonal InTe cluster). The inset of the figure show the lattice parameter decreases as the doping rate increases.

The crystal structure and phase purity of the synthesized Sn_1−x_In_x_Te samples were examined using powder X-ray diffraction, with representative patterns for 0.0 ≤ x ≤ 0.5 shown in Figure 1. All compositions up to x = 0.4 exhibit a single-phase rock-salt structure, and every diffraction peak can be indexed to the cubic Fm-3m space group. The previous investigation reports the mixed phase of InTe in SnTe for the x = 0.4 sample [20], indicating the higher quality single crystal of our sample than that of the previously reported one. For pristine SnTe (x = 0.0), the lattice parameter is a = 6.318 Å, and it decreases linearly with increasing indium content, reaching 6.257 Å at x = 0.5. This monotonic reduction in lattice constant follows Vegard’s law, confirming the successful and uniform substitution of the smaller In atoms for Sn sites throughout the solid solution. At concentrations exceeding x = 0.5, additional reflections corresponding to a tetragonal InTe secondary phase emerge. Consequently, the analysis of superconducting properties in this study is restricted to the single-phase region (x ≤ 0.5) to avoid complications associated with impurity phases. This structural verification establishes a reliable foundation for interpreting the electronic and superconducting behavior induced by systematic indium doping.

We measured the inductively coupled plasma (ICP) spectroscopy to identify the sample concentrations of Sn, In, and Te, which are listed in Table 1. The resulting compositions are almost the same or little bit less than the nominal composition. A smaller indium composition may come from the excess Sn flux during crystal growth.

### 3.2. S-Wave Superconductivity

**Figure 2:**
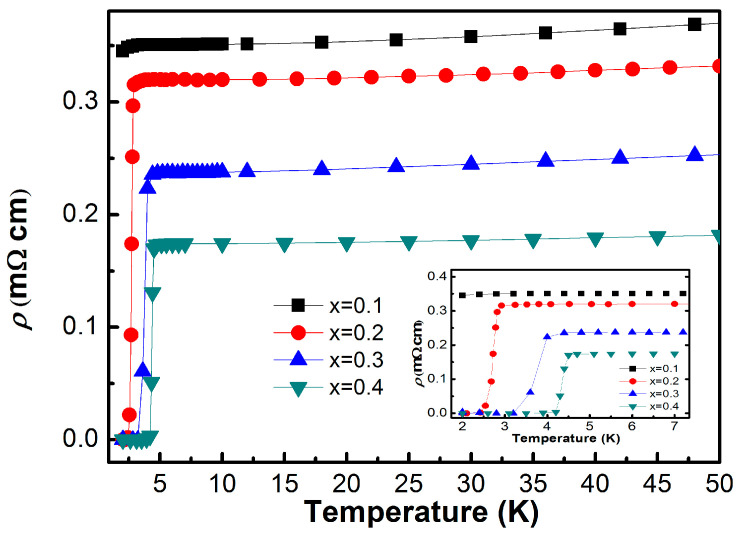
Temperature dependence of the resistivity of Sn_1−x_In_x_Te in zero fields with the x value in the range of x = 0.1–0.4. x = 0.4 shows the highest superconducting transition with $T_{C}$ at ~ 4.3 K. When indium concentration increases, $T_{C}$ increases and overall resistivity decreases.

**Figure 3:**
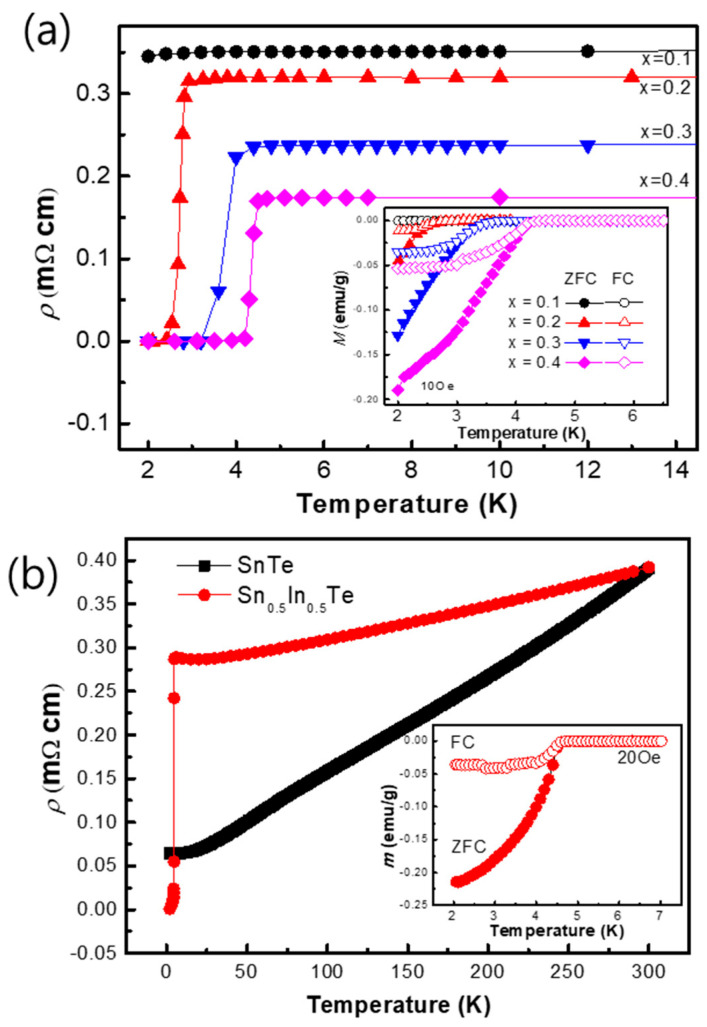
(**a**) The temperature dependence of the resistivity and DC magnetization (inset) of Sn_1−x_In_x_Te under the field-cool (FC, open symbols) and zero-field-cool (ZFC, closed symbols) with the x value in the range of x = 0.0~0.4. When indium concentration increases, *T_c_* increases and resistivity decreases. (**b**) The temperature dependence of the resistivity in a zero magnetic field of SnTe and Sn_0_._5_In_0_._5_Te. The temperature dependence of the magnetization of Sn_0_._5_In_0_._5_Te under the field-cool (FC, red open circles) and zero-field-cool (ZFC, red closed circles) measurements when applying a magnetic field of 20 Oe.

The temperature-dependent resistivity *ρ*(*T*) of Sn_1−x_In_x_Te for x = 0.1–0.4 is presented in Figure 2. All samples display metallic behavior at high temperatures, with resistivity decreasing as the temperature is lowered. At low temperatures, a sharp drop to zero resistivity marks the onset of superconductivity. The superconducting transition temperature T_c_, determined from the peak in *dρ*/*dT*, evolves systematically with increasing In concentration. As shown in the inset of Figure 2 and summarized in Table 2, T_c_ increases monotonically from 1.9 K at x = 0.1 to 4.3 K at x = 0.4. The highest transition temperature, approximately 4.7 K, is observed for the x = 0.5 composition, whose resistivity curve is displayed in Figure 3 and exhibits a clear superconducting transition at *T_c_* ≈ 4.7 K. Concurrently, the normal-state resistivity decreases with higher In content, which can be attributed to an enhanced electronic density of states at the Fermi level, resulting in improved charge transport and strengthened superconducting pairing interactions.

To confirm that the observed superconductivity is a bulk phenomenon, DC magnetization measurements were performed on the Sn_0.5_In_0.5_Te sample in the inset of Figure 3. The zero-field-cooled (ZFC) measurement reveals a strong diamagnetic signal below its $T_{C}$~4.7 K with an estimated magnetization value of −0.22 emu/g at 2 K, which is the definitive signature of the Meissner effect. This result provides unequivocal proof of bulk superconductivity in this material.

The temperature dependence of the resistivity *ρ*(*T*) for several compositions is shown. All samples exhibit metallic behavior at high temperatures, followed by a sharp superconducting transition to zero resistivity at low temperatures. With increasing In concentration, the superconducting transition temperature *T*_*c*_ systematically shifts to higher temperatures, while the transition width remains relatively narrow, indicating bulk superconductivity. The inset highlights the low-temperature region, emphasizing the evolution of *T*_*c*_ and the sharpness of the transitions. Figure 3b displays representative resistivity behavior in an expanded temperature scale, illustrating the normal-state resistivity trend above *T*_*c*_. The resistivity increases monotonically with temperature, and the curvature suggests deviations from simple Fermi-liquid behavior at higher doping levels. The RRR (residual resistivity ratio) values ($R_{300K}/R_{Tc}$) of SnTe and x = 0.5 doped compounds are evaluated as 6.17 and 1.28, respectively, indicating that the impurity scattering dominates in indium rich-substituted samples.

The inset focuses on the vicinity of the superconducting transition, clearly showing the onset of zero resistance. The electrical resistivity and magnetization demonstrates that indium doping enhances superconductivity in Sn_1−x_In_x_Te, increasing *T*_*c*_ while preserving metallic transport in the normal state, and provides transport evidence supporting the doping-induced evolution of superconducting and scattering properties.

**Figure 4:**
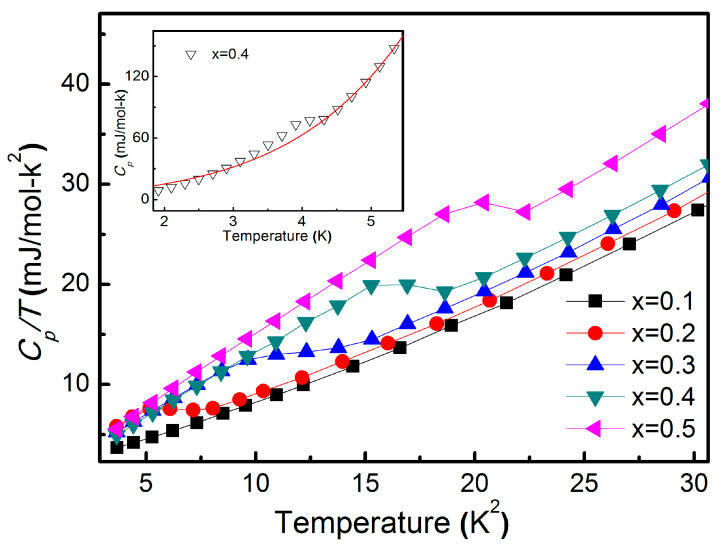
Temperature square (*T*^2^) dependence of the specific heat ($C_{P}/T$) under a zero magnetic field with a composition of x = 0.1–0.5. The inset of the figure denotes the comparison between experimental specific heat (triangle-scattered) with specific heat fitted by $C/T=\gamma +\beta T^{2}+\delta T^{4}$ (red line) at x = 0.4.

To further investigate the bulk nature and thermodynamic characteristics of the superconducting state, we measured the specific heat (*C_p_*) for samples with various indium concentrations. As shown in Figure 4, all superconducting compositions exhibit a clear jump in specific heat at their respective transition temperatures, confirming the presence of bulk superconductivity. To quantify the underlying thermodynamic properties, the normal-state specific heat was fitted using the standard expression $C/T=\gamma +\beta T^{2}+\delta T^{4}$, where *γ*, *β*, and *δ* correspond to the electronic, phononic, and higher-order electron–phonon coupling contributions, respectively [12]. The inset of Figure 4 displays the temperature-dependent specific heat for the x = 0.4 sample; a noticeable deviation between the experimental data (triangle symbols) and the fitted curve (red line) appears around the superconducting transition, consistent with its reported $T_{C}=4.3\;K$. The fitting parameters extracted from this analysis provide important insight into the nature of the pairing mechanism, and their systematic evolution with increasing indium concentration is summarized in Table 2.

**Figure 5:**
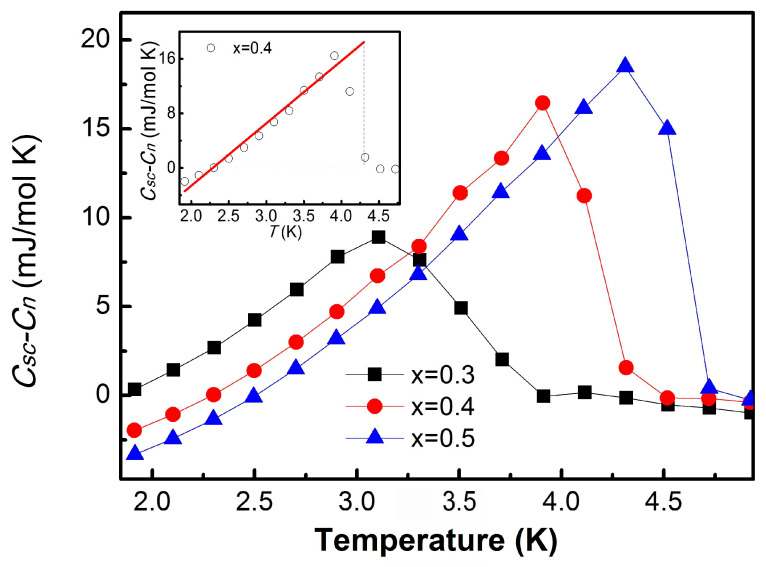
Temperature dependence of the specific heat of the superconducting state subtracted by the specific heat of the normal state ($C_{sc}-C_{n}$). Inset of the figure: the red solid line is linear guide to eye with the experimental value of x = 0.4 and the vertical dot line starts from $T_{C}$.

Figure 5 presents the temperature dependence of the electronic specific heat in the superconducting state, obtained by subtracting the normal-state contribution (*C_sc_ − C_n_*) for the x = 0.3, 0.4, and 0.5 samples. In the background-subtracted data, each composition exhibits a clear anomaly near the superconducting transition, consistent with the corresponding $T_{C}$ values listed in Table 2. The normalized specific heat jump $\Delta C/\gamma T_{C}$ provides an additional measure of coupling strength [26,27]. For x = 0.3, the value $\Delta C/\gamma T_{C}=1.357$ is close to the BCS (Bardeen–Cooper–Schrieffer) weak-coupling limit of 1.43, while higher doping levels show a decrease to 1.009 at x = 0.5. This deviation from the canonical BCS value at high indium concentrations may indicate the limitations of the simple weak-coupling model in the stronger-coupling regime, or it may arise from enhanced impurity scattering associated with the proximity to the solubility limit where InTe secondary phases begin to form.

### 3.3. Weak-to-Strong Coupling Transition by Thermodynamic Properties

**Figure 6:**
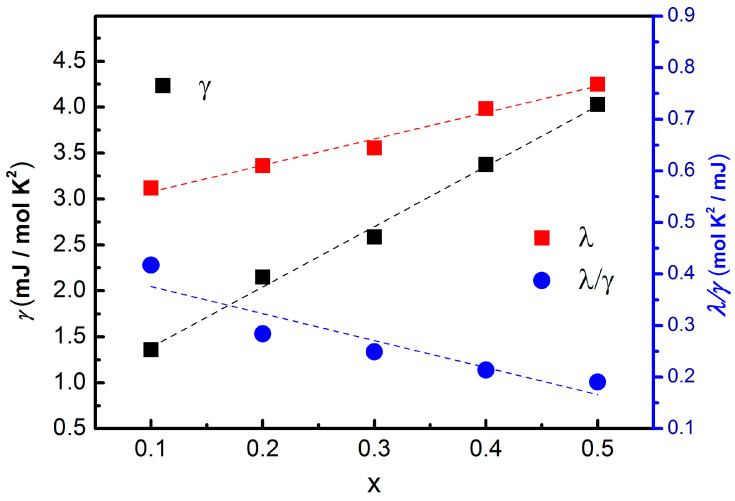
Doping rate dependency of McMillan equation parameters, Sommerfeld coefficient γ, dimensionless electron–phonon coupling constant $\lambda_{el\text{-}ph}$, and effective electron–phonon potential $\lambda_{el\text{-}ph}/\gamma$, respectively. The dashed lines are linear guide to the eye.

A systematic analysis of the extracted thermodynamic parameters reveals several key trends that clarify the evolution of superconductivity in Sn_1−x_In_x_Te. As shown in Table 2, and Figure 6, the Sommerfeld coefficient *γ* increases nearly linearly with indium concentration (black square of Figure 6, left axis). Because *γ* is proportional to the electronic density of states at the Fermi level, this trend indicates that In doping effectively enhances the number of charge carriers available for superconducting pairing.

The electron–phonon coupling constant $\lambda_{el\text{-}ph}$, calculated using the McMillan equation (Equation (1)), also increases monotonically with x, rising from 0.5668 at x = 0.1 to 0.7542 at x = 0.5 (red square of Figure 6, right axis). The Debye temperature $\Theta_{D}$ systematically decreases with increasing indium substitution concentrations. The decrease in the Debye temperature with increasing In concentration originates from lattice softening induced by In substitution, which weakens the average bonding strength and lowers the characteristic phonon energy scale. Therefore, the increase in $T_{c}$ is driven by the combined effects of enhanced electronic density of states and phonon softening, which together strengthen electron–phonon-mediated Cooper pairing because the effective electron–phonon interaction potential $V_{\mathrm{eff}}$ satisfies $\lambda_{el\text{-}ph}=N\left(0\right)V_{\mathrm{eff}}$, where $N\left(0\right)\left(\propto \gamma \right)$ is the density of states on Fermi surface. The simultaneous increase in both γ and $\lambda_{el\text{-}ph}$ is consistent with a phonon-mediated mechanism whose strength is enhanced primarily through an increased density of states.

(1) $$T_{C}=\left(\Theta_{D}/1.45\right)\exp\left[-\frac{1.04\left(1+\lambda_{el\text{-}ph}\right)}{\lambda_{el\text{-}ph}}-\mu^{*}\left(1+0.62\lambda_{el\text{-}ph}\right)\right]$$

where $\mu^{*}$ is for coulomb pseudo-potential $\mu^{*}\sim 0.13$.

**Figure 7:**
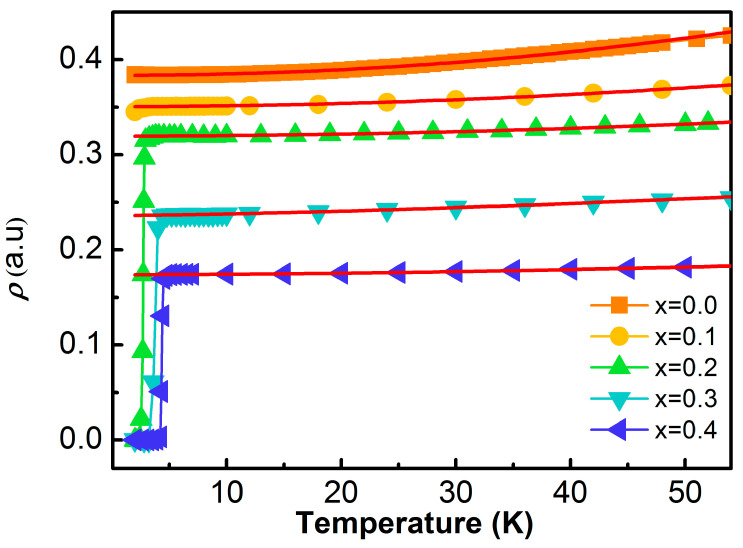
Temperature dependence of the resistivity under a zero magnetic field with an x value in the range of x = 0.0–0.4. We fit the equation $\rho =\rho_{0}+AT^{n}$ (solid red lines); x = 0.0 corresponds to *n* = 2.04 and x = 0.4 to *n* = 1.72.

Further evidence for the evolution of coupling strength is found in the normal-state resistivity behavior. Figure 7 presents the temperature-dependent electrical resistivity with fits to the Fermi-liquid expression $\rho =\rho_{0}+AT^{n}$ revealing that pristine SnTe has an exponent n≈2.04, consistent with typical Fermi-liquid electron–electron scattering. As indium doping increases, the exponent systematically decreases, reaching n=1.72 at x = 0.4. This departure from quadratic temperature dependence signals enhanced scattering processes, which, in a phonon-mediated system with decreasing Debye temperature, point to increasingly strong electron–phonon interactions.

Taken together, the thermodynamic and transport measurements present a coherent picture: indium doping drives the Sn_1−x_In_x_Te system from a weakly coupled to a more strongly coupled superconducting regime. This controlled evolution of coupling strength within a topological crystalline insulator establishes Sn_1−x_In_x_Te as a valuable platform for exploring the interplay between strong electron–phonon interactions, band topology, and unconventional superconducting phenomena.

## 4. Conclusions

In this work, we have synthesized high-quality Sn_1−x_In_x_Te single crystals up to the solubility limit (x ≤ 0.5) and conducted a comprehensive investigation of their structural, transport, and thermodynamic properties. Powder X-ray diffraction confirms that all compositions within this range form a single-phase cubic structure, establishing a reliable platform for evaluating the effects of indium doping on superconductivity.

Our measurements reveal that indium incorporation induces bulk, fully gapped s-wave superconductivity whose transition temperature systematically increases with doping, reaching approximately 4.7 K for x = 0.5. Detailed analysis of the specific heat and McMillan parameters shows that both the Sommerfeld coefficient *γ* and the electron–phonon coupling constant $\lambda_{el\text{-}ph}$ increase monotonically with x, while the Debye temperature remains nearly unchanged. These trends demonstrate that the enhancement of superconductivity originates primarily from electronic effects—most notably an increased density of states at the Fermi level—as well as the lattice softening.

Furthermore, the progressive deviation of the resistivity exponent from the Fermi-liquid value of n=2 provides complementary evidence for strengthened electron–electron interactions or electron-impurity scattering at higher indium concentrations. Together, the thermodynamic and transport data establish a coherent picture of a doping-driven crossover from weak to strong coupling in Sn_1−x_In_x_Te.

These results highlight Sn_1−x_In_x_Te as a tunable superconducting system in which electron–phonon coupling strength can be systematically controlled. Given the material’s intrinsic topological crystalline properties, the demonstrated coupling evolution offers a promising foundation for future studies aimed at uncovering potential topological superconducting phases and exploring the broader interplay between lattice symmetry, electronic topology, and strong-coupling superconductivity.

## Figures and Tables

**Table 1:** Elemental concentrations of Sn, In, and Te and their resulting compositions of Sn_1−x_In_x_Te, characterized by the inductively coupled plasma (ICP) spectroscopy.

Nominal x	Sn (wt.%)	In (wt.%)	Te (wt.%)	Compositions (x)
0.0	48.18	0.0	51.82	0.0
0.1	44.43	3.69	51.88	0.079
0.2	39.87	8.18	51.95	0.175
0.3	35.71	12.27	52.02	0.262
0.4	31.25	16.74	52.11	0.357

**Table 2:** The extracted values from the specific heat measurements (*γ*, *β*, *δ*), transition temperature *T_c_*, Debye temperature *Θ_D_*, electron–phonon coupling constants $\lambda_{el\text{-}ph}$, effective electron–phonon potential ($V_{eff}$), and specific heat jump at transition temperature Δ*C/γT_c_*.

x	*γ* (mJ/mol·K^2^)	*β* (mJ/mol·K^4^)	*δ* (mJ/mol·K^6^)	*T_c_* (K)	*Θ_D_* (K)	*λ_el_* *_-_* *_ph_*	*V_el-ph_* (mol·K^2^/mJ)	Δ*C/γT* *_c_*
0.1	1.359	0.594	0.0089	1.999	186.9	0.5668	0.41716	-
0.2	2.149	0.606	0.0090	2.629	185.6	0.6139	0.28398	1.262
0.3	2.587	0.644	0.0088	3.599	182.1	0.6782	0.24916	1.357
0.4	3.374	0.672	0.0087	4.300	179.5	0.7258	0.21357	1.273
0.5	4.030	0.850	0.0083	4.701	165.9	0.7542	0.19053	1.009

## Data Availability

The original contributions presented in this study are included in the article. Further inquiries can be directed to the corresponding author.
